# P46 Service evaluation of BioFire^®^ Meningitis/Encephalitis panel used at point of care in an acute medical unit

**DOI:** 10.1093/jacamr/dlaf230.053

**Published:** 2025-12-04

**Authors:** Matthew Begbie, Andrew Walden

**Affiliations:** Royal Berkshire Hospital, Reading, UK; Royal Berkshire Hospital, Reading, UK

## Abstract

**Background:**

Meningitis and encephalitis (M/E) are medical emergencies with high morbidity and mortality if not promptly diagnosed and treated. The diagnostic gold standard remains CSF culture via lumbar puncture (LP), but this approach is limited by low sensitivity, long turnaround times and a significant proportion of culture-negative cases despite strong clinical suspicion due to antimicrobial treatment prior to sampling. On the Acute Medical take presentations with suspicion of M/E are common. Delays in CSF sampling and the culture thereof can lead to unnecessary hospital admissions, unnecessary antimicrobial use and increased healthcare costs. Molecular diagnostics such as the BioFire^®^ Meningitis/Encephalitis (ME) panel offer rapid, multiplex PCR-based syndromic detection of 14 common bacterial, viral and fungal pathogens directly from CSF within approximately 1 h. This evaluation sought to assess the performance of the BioFire^®^ ME panel at the point of care (POC) in the Acute Medical Unit (AMU) at Royal Berkshire Hospital, and to consider its impact on clinical workflow during the acute take.

**Methods:**

Over a 19 month period, 105 patients admitted to the acute medical take with suspected meningitis/encephalitis underwent BioFire^®^ ME panel testing alongside conventional LP with CSF analysis and culture. Patient demographics, length of hospital stay and diagnostic outcomes were collected. Pooled patient demographic data was collected and the results of the BioFire^®^ detected organisms and the culture based methods compared.

**Results:**

There were 28 positive cases and 77 negative cases. BioFire^®^ detected pathogens in all 28 positive cases of which 15 were viral and 13 bacterial. CSF culture detected bacterial pathogens from 2 cases. Both CSF-positive cases were confirmed by BioFire^®^ as *Streptococcus pneumoniae*. Five BioFire^®^ tests were invalid; corresponding CSF results were negative. Positive cases mean admission was 16.5 days. Mean admission for viral infections was 13 days and 23 for bacterial infections. *S. pneumoniae* (*n*=6, 21%) was the commonest pathogen, Enterovirus and *Haemophilus influenzae* (*n*=5, 18% each), Varicella zoster virus and Herpes simplex virus 1 (*n*=3, 11% each), Human herpesvirus 6 (HHV-6) and *Neisseria meningitidis* (*n*=2, 7% each) and Herpes simplex virus 2 and *Streptococcus agalactiae* (*n*=1, 4% each). HHV-6 detection may represent latent viral DNA rather than active infection.

**Conclusions:**

The BioFire^®^ ME panel demonstrated markedly superior sensitivity compared with conventional LP culture in this AMU cohort and provided clinically actionable results within the timeframe of the acute medical take. Its rapid turnaround and broad coverage support earlier decision-making, more targeted antimicrobial use and earlier discharge of those with negative results or with viral meningitis. Importantly, the test was feasible to deploy at POC in the AMU, integrating into routine clinical workflow without delaying patient management. While interpretation of certain viral results (e.g. HHV-6) requires caution, this evaluation supports BioFire^®^ as a practical and effective diagnostic tool for meningitis/encephalitis in the acute setting.

